# The role of excitement and enjoyment through subjective evaluation of horror film scenes

**DOI:** 10.1038/s41598-024-53533-y

**Published:** 2024-02-05

**Authors:** Botond László Kiss, Anita Deak, Martina Dominika Veszprémi, Albert Blénessy, Andras Norbert Zsido

**Affiliations:** Institute of Psychology, Faculty of Humanities and Social Sciences, University of Pécs, Ifjúság útja 6, 7624 Pécs, Hungary

**Keywords:** Psychology, Human behaviour

## Abstract

The popularity of the horror genre is constantly increasing and still has not reached its peak. As a recreational activity, people watch horror movies in pursuit of excitement and enjoyment. However, we still do not know what traits make people seek out this type of increase in arousal (excitement) and why they find it desirable (enjoyment). Consequently, in this study, we sought to identify observer-related factors that allows consumption of horror content as a recreational activity. Participants (N = 558) answered questions about movie-watching habits, completed measures of cognitive emotion regulation, sensation seeking, paranormal beliefs, morbid curiosity, disgust sensitivity, and rated short scenes from horror movies on dimensions of excitement, enjoyment, disgust, fearfulness, and realness. Our findings suggest that the predictors of excitement and enjoyment are slightly different. Perceived disgust negatively affected enjoyment but did not influence to excitement. Further, both excitement and enjoyment were positively predicted by fearfulness and realness ratings of the scenes, and morbid curiosity. Paranormal beliefs, sensation seeking, disgust sensitivity, anxiety, and emotion regulation strategies were not associated with excitement and enjoyment. Future studies should make a distinction between excitement and enjoyment as they are equally important factors with slightly different backgrounds in recreational fear.

## Introduction

Even though fear is an unpleasant feeling, fear-related recreational activities, such as haunted houses, horror movies, and video games, are becoming increasingly popular^1–3^. According to Nash Information Services^4^, the market share of horror films (in comparison with other film genres) has grown from 5 to 10% since 1995. Although horror elicits a wide variety of negative feelings, such as fear and disgust, its popularity may lie in the fact that it can also evoke both excitement and enjoyment in the viewer^1,5,6^. Encountering various potential or actual threatening stimuli increases the activity of the sympathetic nervous system resulting in different behavioral, cognitive, and physiological reactions such as increased cardiovascular function and shallow breathing, alertness^7,8^. Supposedly, a fearful event (such as a visit to a haunted house) triggers fear, leading to increased arousal, which can be experienced as excitement. If the observer appraises this excitement as desirable, they will find the event enjoyable. In a recent field study^9^, visitors to a haunted house were equipped with heart rate monitors. They were filmed during the attraction, and after that, they were asked to report their experience. The results show an optimal level of increase in arousal (i.e., excitement caused by fear), where enjoyment is maximized. The term *recreational fear* has been coined to describe the mixed experience of fear, and enjoyment^1^.

There are several theories reflecting the (seemingly) paradox phenomenon where enjoyment and fear coexist. According to Zillman’s^10,11^ excitation transfer conception, the key aspect in the enjoyment of horror is the positive resolution (where either the protagonist achieves a positive outcome or the antagonists a negative one) and the euphoria following the end of the dread. Despite these suggestions, people tend to enjoy movies where the ending no longer focuses on rewarding the protagonist and eliminating the antagonist. An explanation for this tendency could be the benign masochism hypothesis, which states that watching horror movies is an adaptive behavior because it helps us to prepare for threatening events potentially occurring in the future^1^. This is in line with early results that emphasize the pleasure of arousal evoked by horror films as a learned behavior^12^. Although these theories offer an explanation to why certain people watch horror movies, they cannot account for the individual differences determining the appraising process. In fact, these approaches intermix excitement and enjoyment and, hence fail to consider them separately.

There are certain traits that predispose people to find enjoyment in the excitement caused by a fearful setting. The excitement (defined as the increase in arousal) evoked by mild fear can be experienced as rewarding, however, mild fear (similarly to what the optimal arousal level is) is subject to great individual differences^13,14^. Those who are responsive to pleasure induced by fear will seek out different kinds of horror films for satisfaction. People characterized by this behavior are often described as sensation seekers^15^. Sensation-seeking is a personality trait often described by the tendency to search for new, interesting experiences. Past research^16–18^ has shown that sensation-seeking correlates with exposure to horror films, subjects with high interest in horror movies scored higher on sensation-seeking measures. In line with this previously it has been found that morbid curiosity correlates with sensation seeking^19,20^, and, thus, described as a possible origin of morbid curiosity as an individual’s need for stimulation. Morbid curiosity is usually described as a mixture of curiosity, excitement, and fear regarding unpleasant things such as death^21,22^. Scrivner^22^ suggests that we are much more curious about the ways that are leading to death than the death itself. Therefore, another explanation for the great popularity of these films could be that they present morbid and disgusting content. However, although some people find amusement in disgust^23^, the main role of disgust is to protect the self against harmful acts, contaminations, and substances^24^. Perceiving something as disgusting (contrary to threatening) causes parasympathetic activation^25^ which, then, facilitates avoidance behavior. Consequently, if excitement is provoked by disgust, it may decrease enjoyment. Similarly, sensation seeking has shown a negative correlation with disgust sensitivity, suggesting that sensation seekers are more willing to perform different disgusting tasks related to body violation and death^23^. Overall, it seems that while sensation-seeking and morbid curiosity may predispose people to seek excitement and enjoyment in horror content, perceived disgust could do the opposite.

How real the observer perceives the content and how they react to the evoked excitement are also crucial factors determining enjoyment. Increased perceived reality of a situation can influence how exciting people find it. According to the dual awareness theory^26^, the viewer will find the scene more realistic, if an event (e.g., a horror movie scene) can distract them from its fictionality. Beliefs about the world may also influence the perceived reality of a situation. Those who tend to believe more in the supernatural are more likely to perceive paranormal events as real^27^. Furthermore, supernatural threatening elements in horror films trigger the same network as natural threatening elements^28^. This could be because supernatural creatures like vampires and werewolves carry universal characteristics of threat and disgust such as prominent teeth, blood, fur, and salivation^27^. Therefore, initially, the film must seem real, in order to activate the automatic processes that make us experience fear, but later, in the evaluation, it is essential to confirm that it is not real^29,30^. In this evaluation, the process of emotion regulation can be essential. Emotion regulation is a cognitive process by which individuals can influence what emotion they experience in a situation and how it is expressed^31^. Horror movies increase the arousal of the viewer, and people tend to label the evoked feelings mostly negative. Still, if one can adaptively regulate one’s emotions, this excitement can lead to an enjoyable experience Indeed, emotion regulation can play a prominent role in how we label our emotions elicited by the increased level of arousal, e.g., when the protagonist is chased by a maniac killer on the screen. Putatively adaptive strategies (e.g., acceptance, reappraisal) may reduce the negative impact experienced^32^. Previous research showed that reappraisal can reduce the appearance of negative emotions^33,34^, in particular fear and disgust^35,36^. In contrast, putatively maladaptive strategies (e.g., catastrophizing, rumination) may amplify negative effects^32^. That is, horror scenes perceived as being closer to reality may trigger greater excitement (stronger emotions), and regulating the emotions experienced during a horror film could lead to a more enjoyable experience.

The overarching goal of our research was to identify what observer-related or movie-related factors determine the level of enjoyment and excitement of recreational activities that induce fear. We tested the same model for both variables and expected these to be predicted by the level of experienced fear and disgust the scene evokes; perceived realness of the scene, sensation seeking, morbid curiosity, and belief in the supernatural, anxiety and disgust sensitivity and the use of putatively adaptive and maladaptive emotion regulation strategies. Excitement is a process that depends on the elicited level of arousal (sympathetic activation) caused by fear. Enjoyment is influenced through the appraisal of the event; therefore, it is more dependent on disgust as it facilitates avoidance behavior (parasympathetic activation). Consequently, we hypothesized that fear (compared to disgust) would be a stronger predictor of excitement, while disgust (compared to fear) would be a stronger predictor of enjoyment. Overall, we aimed to test the explanatory power of these perceiver-related factors and to present a possible conceptual model.

## Material and methods

### Participants and procedure

The required minimum sample size for this study was determined by computing estimated statistical power with a conservative approach (f = 0.1, power = 0.95, α = 0.05, df = 11) using the pwr package for R^37,38^. The analysis indicated a required total sample size of 260. A total of 593 (442 female) people participated in the study, 35 participants were excluded from the analyses as they did not agree to watch the videoclips. Thus, the analyses were conducted with 558 (413 female) participants (age range: 18–76, mean age = 34.2, SD = 10.6). For more detailed features of the sample see Table 1. The research was approved by the Hungarian United Ethical Review Committee for Research in Psychology (reference number: 2022-05) and was carried out following the Code of Ethics of the World Medical Association (Declaration of Helsinki). Informed and written consent was obtained from all participants.

**Table 1:** Demographic characteristics of the sample by gender, watching horror movies, horror liking, preferred horror genres, and the questionnaires used in the study.

Variable	Category	Count	
Gender	Female	413	
	Male	145	
Watching horror	Yes	451	
	No	107	
Liked horror	Yes	431	
	No	48	
	Cannot decide	79	
Preferred horror genre	Psychological	386	
	Gore	98	
	Killer	282	
	Monster	175	
	Paranormal	223	

	Mean	Median	SD
STAI	12.8	13.0	4.25
MCS	93.8	93.0	23.4
PBS	53.4	53.0	24.7
DSR core	0.835	0.833	0.304
BSSS	24.1	24.0	7.01
aER	34.1	35.0	6.66
maER	22.5	23.0	6.13

*STAI* State-Trait Anxiety Inventory, *MCS* Morbid curiosity Scale, *PBS* Paranormal Belief Scale, *DS-R core* Disgust Scale Revised Core subscale, *BSSS* Brief Sensation Seeking Scale, *aER* Adaptive Emotion Regulation strategies, *maER* Maladaptive Emotion Regulation strategies.

We collected data online with the help of Google Forms, during the spring of 2021. The questionnaire was distributed via various movie-themed social media groups and e-mail lists. First, participants completed sociodemographic and horror movie-related questions. Then they filled out questionnaires assessing anxiety, morbid curiosity, supernatural belief, disgust sensitivity, sensation-seeking, and emotion regulation strategies. After answering the questions, the participants could move on to watch the horror movie scenes. If they agreed and moved on, all participants could view the film scenes in the same order. After rating (along the five dimensions) a given film scene, they could move on to the next scene. It took approximately 30 min to complete the survey.

### Questionnaires

We used the 18-item version of the *Cognitive Emotion Regulation Questionnaire* (CERQ)^39^ to assess the participants' emotion regulation strategies. The scale measures putatively adaptive emotion regulation (aER) strategies across five subscales (Putting into Perspective, Positive Refocusing, Positive Reappraisal, Acceptance, Planning) and putatively maladaptive emotion regulation (maER) strategies across four subscales (Self-blame, Other-blame, Rumination, Catastrophizing). The higher the score of individual subscales the more that specific cognitive strategy is used. The subscale reliability values were acceptably high for the CERQ-short subscales (McDonald’s ꞷ for aER was 0.782; and 0.821 for maER).

To measure individuals Sensation seeking predisposition we used the *Brief Sensation Seeking Scale* (BSSS) consisting of eight items^40^. Zuckerman^21^ identified four dimensions of sensation seeking included in the sensation seeking scale: thrill and adventure seeking, experience seeking, disinhibition, and boredom susceptibility. Participants scoring higher on the scale have a higher tendency to participate in different sensation-seeking activities and behaviors. The results of our reliability analysis for the BSSS were good (McDonald’s ꞷ = 0.795).

The Revised Paranormal Beliefs Scale^41^ was used to assess individuals' belief in paranormal phenomena. The scale measures paranormal beliefs in different dimensions: Traditional Religious Belief, Psi, Witchcraft, Superstition, Spiritualism, Extraordinary Life Forms, and Precognition. People who score higher on the scale are more likely to believe in certain supernatural notions. For redaction purposes, we decided to remove two scales that are irrelevant to our study. The results of our reliability analysis showed very high reliability (McDonald’s ꞷ = 0.944).

We measured morbid curiosity with the help of the *Morbid Curiosity Scale* (MCS) consisting of 24 items^22^. The scale consisted of 24 items distributed across four subscales: Violence, Body Violation, Motives of Dangerous People, and Supernatural Danger. Individuals who score higher on the scale are more likely to experience curiosity or excitement in connection with unpleasant topics. The results of our reliability analysis were highly acceptable (McDonald’s ꞷ = 0.904).

Disgust sensitivity was measured using the core disgust subscale of the Revised Disgust Scale (DS-R)^42^. The subscale measures general disgust proneness. Individuals with higher scores are more likely to experience disgust and are more sensitive to disgust-inducing content. Our data showed good scale reliability (McDonald’s ꞷ = 0.731).

We measured trait anxiety with the short, five item version of the *Spielberger State-Trait Anxiety Inventory* (STAI)^43^. STAI measures trait anxiety (how one feels in general). Higher scores indicate higher trait anxiety levels. The reliability of the scale on our sample was very good (McDonald’s ꞷ = 0.861).

### Video material

The video material comprised short clips from major horror sub-genres. This was necessary because we wanted to get an overall picture of the motives of horror consumption that may be generalized to all sub-categories. Participants watched 10 short horror films, two each from five genres, such as killer, psychological, body and gore, supernatural, and monster. We provide more details on the characterization of these sub-genres in Table 2. The video material was pre-selected by 15 independent participants. During the pre-selection, the participants were asked to choose which of the five categories they thought the scene fit into. We selected the scenes that 90% of the participants put in the same category.

**Table 2:** A detailed description of each of the five horror film genres (Gore, Killer, Monster, Paranormal and Psychological) we have selected.

Horror movie genre	Description
Gore	Gore focuses on vividly depicting graphic violence, featuring classic elements like blood, guts, and body trauma
Killer/Slasher	Killer/Slasher horror features a killer (human-like character, sometimes with supernatural abilities) who hunts down a certain group of people. The hunt for the members of the group usually ends in a uniquely bloody, brutal murder, which is a key element of the film
Monster	In monster horror movies, a wide variety of creatures (from zombies to werewolves) cause destruction in a certain pattern
Paranormal	Paranormal horror revolves around non-living entities like spirits and ghosts that evoke fear through unexplained occurrences
Psychological	Psychological horror revolves around evoking emotions rather than visual elements, playing with viewers' minds to create paranoia and influencing their emotional states

Each scene lasted around 20–30 s. After each scene, they were asked to rate the horror film scenes on five dimensions—fearfulness, realness, disgust, enjoyment, and excitement. Ratings were based on a 5-point Likert scale (1 = not at all, 5 = very much). We asked participants to rate the scenes according to how they felt while watching them. We also asked them whether they had seen that scene earlier. See Table 3 for the central tendencies of the ratings. The detailed descriptions (title, year, director, studio, genre, timestamp) of each used scene are presented in Supplementary Material 1.

**Table 3:** Means (M) and standard deviations (SD) for the horror film scenes ratings on dimensions of fearfulness, realness, disgust, enjoyment, and excitement presented by genre. Values range from 1 to 5 with higher values indicating higher ratings.

Genre	Excitement		Enjoyment		Disgust		Fearfulness		Realness	
	Mean	SD	Mean	SD	Mean	SD	Mean	SD	Mean	SD
Monster	3.12	0.98	2.95	1.12	2.07	0.98	2.57	1.03	2.22	1.03
Killer	2.85	1.08	2.53	1.17	2.69	1.13	2.57	1.13	2.95	1.13
Gore	2.66	1.19	2.32	1.20	3.50	1.24	2.36	1.18	2.60	1.06
Psychological	3.83	1.02	2.96	1.14	1.32	0.62	2.73	1.17	3.27	1.16
Paranormal	4.09	0.96	3.62	1.22	1.46	0.78	3.80	1.13	3.15	1.27

### Statistical analysis

For the statistical analyses, we used Jamovi software version 2.3.18^44^. To assess the internal reliability of the questionnaires, we used McDonald’s ꞷ^45^. The assumption of normality was not violated, the absolute value of Skewness and Kurtosis were less than 2 for all variables used^46^.

We used *General Linear Modelling* (GLM) to test what measured variables were significant predictors of the enjoyment and excitement rating. We tested two models in total, separately for enjoyment and excitement ratings with the summary scores as dependent variables (DV). We decided to use the summary scores because we wanted to get an overall picture of the motives of horror consumption. Further, the design of the study (i.e., the number of clips per category) precluded the estimation of interactions of genre and type of emotional response. In both models, the independent predictors were disgust, fearfulness and realness ratings, maladaptive and adaptive emotion regulation, disgust sensitivity, paranormal beliefs, morbid curiosity, and anxiety independent predictors and horror-watching Multicollinearity were not a concern in the models (VIF values were below 4). See Supplementary Table 2 to see correlational coefficients across all included variables.

## Results

### General linear models

To explore what measured factors determine how people perceive horror scenes, we used GLMs. Table 4 shows the detailed statistical results. Overall, both models explained a high percentage of the variance (adj. R^2^_excitement_ = 0.456, adj. R^2^_enjoyment_ = 0.416). The results of the GLM analysis show that both the enjoyment and excitement ratings of the scenes were predicted positively by fearfulness and reality ratings, by the level of morbid curiosity, and by whether they watch horror or not. While enjoyment ratings were also predicted negatively by the disgust ratings. The results of the GLM only partially supported our hypothesis as emotion regulation, paranormal beliefs, disgust sensitivity, sensation seeking, and anxiety did not appear as significant predictors in the models.

**Table 4:** Results of the GLM analysis, the presented values are standardized estimates (β) and the 95% confidence interval.

Predictors and factors	Dependent variable					
	Excitement (Model 1)	CI 95%		Enjoyment (Model 2)	CI 95%	
		Lower	Upper		Lower	Upper
Disgust	− 0.06	− 0.152	0.032	− 0.172*	− 0.267	− 0.076
Fearfulness	0.516*	0.422	0.610	0.217*	0.120	0.315
Realness	0.23*	0.155	0.305	0.341*	0.174	0.329
aER	0.036	− 0.024	0.107	0.038	− 0.015	0.120
maER	0.041	− 0.041	0.112	0.053	− 0.042	0.117
DSR core	− 0.011	− 0.078	0.055	− 0.051	− 0.121	0.018
PBS	− 0.024	− 0.091	0.043	− 0.023	− 0.093	0.046
MCS	0.147*	0.070	0.224	0.233*	0.153	0.313
BSSS	0.038	− 0.033	0.108	0.072	− 0.001	0.145
STAI	0.062	− 0.015	0.139	0.057	− 0.023	0.136
Watching horror	0.446*	0.280	0.611	0.876*	0.704	1.048
Adjusted R^2^	0.456			0.416		
F(11,546)	43.5*			37.05*		

*maER* maladaptive emotion regulation, *aER* adaptive emotion regulation, *DSR core* Disgust Scale Revised core subscale, *PBS* paranormal Belief Scale Revised, *MCS* Morbid Curiosity Scale, *BSSS* Brief Sensation Seeking Scale, *STAI* State-Trait Anxiety Inventory Short version.

*p < 0.001.

## Discussion

Horror stories have been told for centuries despite the aversive emotions, such as fear and disgust, they evoke. Recreational activities that involve fear are becoming increasingly popular in recent times (e.g., horror movies, haunted houses, etc.)^1–3^. Past research has tried to explain the possible theoretical background to why some individuals enjoy these fear and sometimes disgust-inducing activities^1,10,11^. A recent study found physiological evidence that some people tend to enjoy a certain level of fear^9^. However, the distinction between excitement and enjoyment has not yet been made. Furthermore, it is still not clear what factors make an otherwise terrifying event enjoyable and exciting. Thus, we aimed to investigate how the perceived characteristics of the scene (reality, fear, disgust) and personal factors (sensation seeking, disgust sensitivity, morbid curiosity, anxiety, and supernatural beliefs) play a role in the excitement and enjoyment of horror movies. According to our results, the level of excitement was influenced by the perceived fearfulness and realness and the level of morbid curiosity. While the level of enjoyment was related positively to the perceived fearfulness and realness and the level of morbid curiosity and negatively to the perceived disgust.

Perceived disgust negatively affected enjoyment but did not influence excitement. That is, scenes that were found more disgusting were rated as less enjoyable. This may be because disgust has the opposite effect of increased arousal, for example by lowering the heart rate. Our results show that the more disgusting a scene was perceived to be, the less enjoyable they found it. This is in line with the disease avoidance hypothesis^24^. Disgust is an aversive emotion that plays a role in avoiding potential contamination. The possibility of contamination has been associated with several different cues, which can appear in horror films, through blood, mutilation, or the external features of various monsters^17,23^. Therefore, the content is just as important as the level of excitement it evokes in determining how enjoyable one finds it, possibly due to the difference in the behavioral characteristics associated with the sympathetic and parasympathetic nervous systems.

Overall, we found that higher levels of perceived fearfulness, reality, and morbid curiosity can cause higher excitement and enjoyment in the case of horror. Scenes that participants perceived as more real and fearful were more exciting and enjoyable, possibly because they were causing a greater increase in arousal. This increase in arousal can create more excitement and according to the reappraisal theory^13,14^, can have an effect on how much the viewer enjoys the given scene. This can be also linked to the results of the field study by Andersen and colleagues^9^. The perception of reality can be a predictor, i.e., if we are more willing to evaluate such scenes as more real, we can more easily become involved in them^27^, and thus more easily affect us emotionally. Further, in line with the concept of morbid curiosity^19,20,22^, participants who were more curious about morbid events found the scenes more enjoyable and exciting. This is in line with past studies showing that morbid curious people tend to seek out gruesome content to feel excited and find amusement topics related to death^22^. These findings highlight the importance of both excitement and enjoyment.

We did not find any evidence that emotion regulation (adaptive or maladaptive), sensation seeking, anxiety, paranormal beliefs, or disgust sensitivity directly affects the perception of excitement and enjoyment in horror content. Although some recent studies suggest that emotional coping skills could be important during recreational fear activities^6,47^, there are also numerous studies suggesting the role of sensation-seeking in the case of recreational fear activities^19,20,22,48^. Interestingly, while we did find a positive correlation between these factors (see Supplementary Material 2), their effects were nonsignificant in the GLM model. It might be possible that sensation seeking does not directly affect excitement/enjoyment, only indirectly through other relevant factors. In fact, in horror films, like in morbid curiosity, the focus is not necessarily on death (the sensation or thrill), but rather on the way to it (see benign masochism hypothesis). Since both disgusting and paranormal content often appears in horror films, it would be logical to assume that sensitivity or openness to such content also affects the level of excitement and enjoyment. However, we did not find evidence of the effect of these factors.

Some limitations of the present should be noted. Even though we distributed the survey on various platforms, most of the participants reported that they like horror movies and watch horror content regularly, overall, the sample of our study is not representative of the Hungarian population. A more balanced sample may provide better support for our findings. It is also important to note that our findings are based on self-report data. In further research, physiological measurements such as electrodermal activity and electrocardiogram should be applied for more accurate results in perceived excitement and enjoyment. Furthermore, we used relatively short videos as visual stimuli. Longer ones might elicit more intense, longer-lasting emotions from participants, which would help to improve the accuracy of the measurements. Moving forward, it may also be worthwhile to use questions that ask about the reasons or considerations for which participants watch horror movies. Finally, it shall also be noted that the descriptive statistics show a great variability in the excitement and enjoyment ratings of the sub-genres. The goal of the present study was to investigate what traits are associated with the liking of the horror genre in general. Although the differences in the mean values do not necessarily indicate a difference in the pattern of the predictor variables (or individual differences within the participants), it would be interesting to explore the different features of the sub-genres and to investigate if, sub-genre could be moderator of the obtained associations. Unfortunately, the design of the present study (e.g., number of clips per category) precluded the estimation of interactions of genre and type of emotional response. Therefore, it remains an open avenue for future research to discover.

## Conclusions

In sum, our results suggest that excitement and enjoyment of horror-related content are not intermixable, as previous studies used them. In fact, somewhat different factors affect them, and they may be worth investigating separately in the future. We proposed a possible framework to explain why some people tend to enjoy or get excited about watching horror content. Our results underscore the importance of perceived fearfulness, disgust, reality, and morbid curiosity in the subjective perception of threatening content. These results correlate with previous studies that have also investigated horror content viewing or exposure to threatening content. Our findings can help deepen our understanding of emotional reactions toward threatening content whether it is encountered voluntarily or involuntarily. In addition to this, both recreational fear and morbid curiosity are emerging concepts in the field of psychology. A more detailed exploration of these two phenomena may contribute to a more precise understanding of fear and the paradoxical feature of horror.

### Supplementary Information

Supplementary Information 1.

Supplementary Information 2.

## Data Availability

Data are available from the corresponding author upon request.
