# Supplementary material for: The role of excitement and enjoyment through subjective evaluation of horror film scenes
Source: Sci Rep. 2024 Feb 5;14:2987. doi: 10.1038/s41598-024-53533-y (PMC10844225; doi:10.1038/s41598-024-53533-y)
Supplement: Supplementary file 1 — Supplementary Information 1. [file 41598_2024_53533_MOESM1_ESM.docx]

**Supplementary Material 1 – Video material**

Here we provide a detailed description of the 10 horror movie scenes used in our study. In the tables below we indicate the title of the movie, the release year, the director's name, the producing film studio, and the exact time stamps of start and end date (hour: minute: second) of the scenes that we used from each movie.

|  | Video 1 | Video 2 | Video 3 | Video 4 | Video 5 |
| --- | --- | --- | --- | --- | --- |
| Movie title | Dawn of the dead | Halloween | The ruins | Pan's Labyrinth | Texas Chainsaw 3D |
| Release year: | 2004 | 2018 | 2008 | 2006 | 2013 |
| Director: | Zack Snyder | David Gordon Green | Carter Smith | Guillermo del Toro | John Luessenhop |
| Studio: | Strike Entertainment | Miramax | Red Hour Films | Warner Bros Pictures | Lionsgate |
| Genre: | Monster | Killer | Gore | Monster | Killer |
| Scene from the movie: | 00:07:36-00:07:57 | 00:48:10-00:48:25 | 01:01:22-01:01:36 | 00:59:53-01:00:17 | 00:42:16-00:42:229 |
| Excitement | 3.33 | 2.94 | 2.83 | 2.91 | 2.77 |
| Enjoyment | 2.86 | 2.69 | 2.33 | 3.05 | 2.36 |
| Disgust | 1.82 | 2.00 | 3.75 | 2.31 | 3.39 |
| Fearfulness | 2.63 | 2.52 | 2.44 | 2.52 | 2.63 |
| Realness | 2.49 | 3.22 | 3.33 | 1.94 | 2.68 |
| Saw it before (%) | 36.2 | 34.2 | 22.2 | 45.9 | 43.5 |

|  | Video 6 | Video 7 | Video 8 | Video 9 | Video 10 |
| --- | --- | --- | --- | --- | --- |
| Movie title | Black Swan | The Conjuring 2 | Hatchet 3 | Lights out (short movie) | The Blair Witch Project |
| Release year: | 2010 | 2016 | 2013 | 2013 | 1999 |
| Director: | Darren Aronofsky | James Wan | B. J. McDonnell | David F. Sandberg | Daniel Myrick & Eduardo Sánchez |
| Studio: | Fox Searchlight Pictures | Warner Bros Pictures | Dark Sky films | Sandberg Company | Artisan Entertainment |
| Genre: | Psychological | Paranormal | Gore | Paranormal | Psychological |
| Scene from the movie: | 01:20:22-01:20:40 | 00:47:17-00:47:48 | 01:13:30-01:13:54 | 00:00:12-00:00:36 | 00:44:25-00:44:50 |
| Excitement | 3.52 | 4.04 | 2.49 | 4.14 | 2.92 |
| Enjoyment | 3.52 | 3.62 | 2.30 | 3.62 | 2.60 |
| Disgust | 1.36 | 1.60 | 3.26 | 1.32 | 1.27 |
| Fearfulness | 2.78 | 3.70 | 2.27 | 3.91 | 2.68 |
| Realness | 3.03 | 2.84 | 1.88 | 3.46 | 3.51 |
| Saw it before | 31.2 | 63.1 | 17.9 | 40.7 | 57.5 |
