# Supplementary material for: The role of excitement and enjoyment through subjective evaluation of horror film scenes
Source: Sci Rep. 2024 Feb 5;14:2987. doi: 10.1038/s41598-024-53533-y (PMC10844225; doi:10.1038/s41598-024-53533-y)
Supplement: Supplementary file 2 — Supplementary Information 2. [file 41598_2024_53533_MOESM2_ESM.docx]

**Supplementary Material 2 – Correlation matrix**

In the following table we provided the result of the Pearson correlations between the investigated variables, such as the video rating aspects (Enjoyment, Excitement, Disgust, Realness, and Fearfulness) *STAI* State Trait Anxiety Inventory, *MCS* Morbid Curiosity Scale, *PBS* Paranormal Belief Scale, *DS-R core* Disgust Scale Revised Core subscale, *BSSS* Brief Sensation Seeking Scale, *aER* Adaptive Emotion Regulation strategies, *maER* Maladaptive Emotion Regulation strategies. We presented Pearson’s r and the p-value for each variable.

| Variable |  | Enjoyment | Excitement | Disgust | Realness | Fearfulness | BSSS | DSR core | MCS | PBS | STAI | aER |
| --- | --- | --- | --- | --- | --- | --- | --- | --- | --- | --- | --- | --- |
| Enjoyment | Pearson’s r |  |  |  |  |  |  |  |  |  |  |  |
|  | p |  |  |  |  |  |  |  |  |  |  |  |
| Excitement | Pearson’s r | .678 |  |  |  |  |  |  |  |  |  |  |
|  | p | <.001 |  |  |  |  |  |  |  |  |  |  |
| Disgust | Pearson’s r | -.107 | .278 |  |  |  |  |  |  |  |  |  |
|  | p | <.011 | <.001 |  |  |  |  |  |  |  |  |  |
| Realness | Pearson’s r | .375 | .518 | .302 |  |  |  |  |  |  |  |  |
|  | p | <.001 | <.001 | <.001 |  |  |  |  |  |  |  |  |
| Fearfulnes | Pearson’s r | .148 | .556 | .673 | .522 |  |  |  |  |  |  |  |
|  | p | <.001 | <.001 | <.001 | <.011 |  |  |  |  |  |  |  |
| BSSS | Pearson’s r | .261 | .103 | -.136 | .038 | -.104 |  |  |  |  |  |  |
|  | p | <.001 | .015 | .001 | .372 | .014 |  |  |  |  |  |  |
| DSR core | Pearson’s r | -.082 | .059 | .300 | .029 | .173 | -.123 |  |  |  |  |  |
|  | p | .053 | .168 | <.001 | .494 | <.001 | .004 |  |  |  |  |  |
| MCS | Pearson’s r | .408 | .174 | -.343 | .093 | -.151 | .447 | -.170 |  |  |  |  |
|  | p | <.001 | <.001 | <.001 | .028 | <.001 | <.001 | <.001 |  |  |  |  |
| PBS | Pearson’s r | .173 | .161 | .078 | .188 | .119 | .212 | .078 | .286 |  |  |  |
|  | p | <.001 | <.001 | .065 | <.001 | .005 | <.001 | .066 | <.001 |  |  |  |
| STAI | Pearson’s r | .107 | .148 | .144 | .070 | .091 | .052 | .217 | .081 | .148 |  |  |
|  | p | .012 | <.001 | <.001 | .101 | .032 | .222 | <.001 | .057 | <.001 |  |  |
| aER | Pearson’s r | .157 | .179 | .087 | 0.136 | .153 | .160 | .018 | .109 | .065 | -.001 |  |
|  | p | <.001 | <.001 | .040 | <.001 | <.001 | <.001 | .669 | .010 | .123 | .990 |  |
| maER | Pearson’s r | .090 | .149 | .147 | .050 | .131 | -.027 | .144 | .020 | .002 | .547 | .197 |
|  | p | .033 | <.001 | <.001 | .239 | .002 | .522 | <.001 | .645 | .961 | <.001 | <.001 |
